# α-Parvin Expression in Breast Cancer Tissues: Correlation with Clinical Parameters and Prognostic Significance

**DOI:** 10.3390/cells13181572

**Published:** 2024-09-19

**Authors:** Midori Takeda, Hiroaki Ito, Keisuke Kitahata, Sota Sato, Akira Nishide, Kanae Gamo, Shunsuke Managi, Tohru Tezuka, Akihiko Yoshizawa, Minsoo Kim

**Affiliations:** 1 Laboratory of Integrative Molecular Medicine, Graduate School of Medicine, Kyoto University, Yoshida-konoe-cho, Sakyo-ku, Kyoto-shi 606-8501, Kyoto, Japan; takeda.midori.102@m.kyushu-u.ac.jp (M.T.);; 2 Urban Institute & Department of Civil Engineering, Kyushu University, 744 Motooka, Nishi-ku, Fukuoka-shi 819-0395, Fukuoka, Japan; 3 Department of Diagnostic Pathology, Kyoto University Hospital, 54 Shogoin Kawahara-cho, Sakyo-ku, Kyoto-shi 606-8507, Kyoto, Japan; 4 FIMECS, Inc., 26-1, Muraoka-Higashi 2-chome, Fujisawa-shi 251-0012, Kanagawa, Japan; 5 Department of Diagnostic Pathology, Nara Medical University, 840 Shijo-cho, Kashihara-shi 634-8521, Nara, Japan

**Keywords:** α-parvin, stroma, breast cancer, HALO

## Abstract

Stromal cells play a critical role in the tumor microenvironment of breast cancer (BC), as they are recruited by tumor cells and regulate the metastatic spread. Though high expression of α-parvin, a member of the parvin family of actin-binding proteins, is reported to be associated with a poor prognosis and metastasis in several cancers, its role in carcinogenesis has not been thoroughly explored. Therefore, we aimed to examine the expression of α-parvin in BC patients by compartmentalizing and quantifying tissues to determine whether α-parvin can be a potential therapeutic target. We performed immunohistochemical (IHC) staining of α-parvin in BC tissues, and the IHC scores were calculated in the overall tissue, stroma, and epithelium using image analysis software. The expression of α-parvin was significantly higher in BC tissues (*p* = 0.0002) and BC stroma (*p* < 0.0001) than in normal tissues. Furthermore, all α-parvin scores were significantly positively correlated with the proliferation marker Ki67. The overall and stroma scores are associated with the tumor, (lymph) node, and metastasis (TNM) classification, stage, and grade. These results suggest that high expression of α-parvin in stroma is associated with BCs and might be a new predictive marker for diagnosing BC.

## 1. Introduction

Cancer is one of the leading causes of death worldwide. It is estimated that there will be 19.3 million new cases and 10 million deaths due to cancer in 2020 [1]. In 2020, BC was the most common cancer worldwide, with 2.26 million cases diagnosed [1]. BC is classified according to the presence or absence of hormone receptors (ER: estrogen receptor and PR: progesterone receptor) and human epithelial receptor 2 (HER2) to determine the treatment. Hormone receptor-positive BC, HER2-positive BC, and triple-negative BC (TNBC: ER-, PR-, and HER2-) have been categorized and treated with respective targeted medicines in recent years [2,3,4]. The introduction of targeted therapies has markedly improved the prognosis of hormone receptor-positive and HER2-positive BC. In contrast, a worse prognosis has been reported in TNBC than any of the other clinical subtypes [5,6,7]. TNBC accounts for 12~20% of BC patients [6,8,9], and surgical treatment, chemotherapy, and radiation therapy are applied due to the lack of targeted therapies. The discovery of further molecular targets for BC is highly anticipated to improve its prognosis. 

Despite these substantial breakthroughs in treatment, BC eventually leads to distant metastasis in 30–40% of patients, which is the primary cause of BC-related deaths [10]. In several cancers, including BC, stromal cells contribute to metastatic spread by being involved in all stages of extracellular matrix (ECM) remodeling, invasion, and epithelial-mesenchymal transition (EMT) induction [11,12]. Identifying the target proteins for BC in stromal cells might therefore lead to the development of novel treatment and diagnostic tools. 

BC progression and metastasis are regulated by multiple signaling pathways related to proliferation, cell death, differentiation, motility, and interactions with the ECM [13]. Proteins involved in these cellular processes may be potential therapeutic targets. The PARVA gene encodes α-parvin, which localizes to cell adhesion sites and regulates the actin cytoskeleton and cell survival [14,15,16,17]. α-Parvin assembles in a complex with integrin-linked kinase (ILK) and particularly interesting Cys-His-rich protein (PINCH), forming the IPP (ILK/parvin/PINCH) complex, and plays a crucial role in the integrin-mediated signaling [14,16,17,18]. There are reports that α-parvin promotes cancer progression and metastasis in BC and lung adenocarcinoma [19,20,21], while downregulation of α-parvin promotes metastasis in prostate cancer [22]. Sun et al. [21] showed high expression of α-parvin in BC tissues, but the expression levels were only assessed in overall BC tissues.

This study aimed to provide an objective quantitative evaluation of α-parvin levels in different compartments of BC tissue in patients, using image analysis software as well as a detailed analysis of its expression in all BC subtypes. We also explored the possibility of α-parvin being a novel BC biomarker through correlation analysis with BC indices.

## 2. Materials and Methods

### 2.1. In Silico Analysis

cBioPortal (http://www.cbioportal.org (accessed on 1 August 2024)) was used to analyze PARVA gene amplification across human cancers. A meta-analysis of overall patient survival was conducted utilizing the online platform Kaplan–Meier plotter, available at http://kmplot.com/analysis/ (accessed on 1 August 2024) [23,24]. Patients were stratified into two cohorts, characterized by low and high α-parvin protein expression determined through the auto-select best cutoff method [25]. The graphs display the patient counts in the low- and high-expression groups and *p*-values. Overall survival comparisons were performed using the log-rank test, and the adjusted hazard ratio (HR) with a 95% confidence interval (CI) was calculated using a Cox proportional hazards model. A dataset from human BC cell lines from the Cancer Dependency Map (DepMap, https://depmap.org/portal/ (accessed on 1 August 2024)) database was used for correlation analysis between PARVA gene amplification (Copy Number Public 23Q2) and RNA expression (Expression Public 23Q2). 

### 2.2. Cell Culture

Human BC cell lines MDA-MB-231, MDA-MB-468, MCF-7, and BT-474 were maintained in high-glucose Dulbecco’s modified Eagle’s medium (Nacalai Tesque Inc., Kyoto, Japan), supplemented with 10% fetal bovine serum (FBS) and 100 units/mL penicillin and 100 μg/mL streptomycin at 37 °C and 5% CO_2_. MDA-MB-436 and MDA-MB-453 were maintained in L15 medium with 10% FBS, 100 units/mL penicillin, and 100 μg/mL streptomycin at 37 °C without CO_2_.

### 2.3. Immunoblotting (IB) Analysis

Cells were lysed with the lysis buffer (50 mM Tris-HCl, pH 7.6, 1% (*v*/*v*) NP-40, 150 mM NaCl, 1 mM EDTA) and subjected to IB. α-Parvin (cat no. 8190, Cell Signaling Technology, MA, USA) and glyceraldehyde-3-phosphate dehydrogenase (GAPDH) (cat no. 016-25523, FUJIFILM Wako Pure Chemical Corporation, Osaka, Japan) antibodies were used as primary antibodies. The signals were visualized using Chemi-Lumi One Ultra (cat no. 11644-40, Nacalai Tesque Inc.) and detected with a Chemi-doc (BioRad, Hercules, CA, USA). Quantification was carried out using Image Lab software (Ver. 6.1, Bio-Rad).

### 2.4. Tissue Microarray and Patient Information

Paraffin-embedded human BC tissue microarray (TMA) slides were purchased from TissueArray.com LLC (Derwood, MD, USA). BC tumor microarrays BR251f and BC081116e were used for this study. The associated data of BC081116e, including immunohistochemistry (IHC) (ER: estrogen receptor/PR: progesterone receptor/HER2: human epidermal growth factor receptor 2/Ki67) information, pathology grade, and TNM/Stage (AJCC 7th edition), was downloaded from the manufacturer’s website. Since the company provided no information about the individuals, it is not disclosed what treatment each individual received and how they chose to receive it. As per TissueArray.com LLC, the acquisition of all tissues adhering to the utmost ethical standards ensured donors were thoroughly informed and provided their consent. Protocols were in place to uphold standard medical care and safeguard donor privacy, and all human tissue collection followed approved Health Insurance Portability and Accountability Act (HIPAA) protocols.

### 2.5. Study Design

This study was a cross-sectional study, and the case selection criteria were that one TMA slide contained at least ten cores of each clinical subtype (HR [hormone receptor: ER and PR]+, HER2+, HR + HER2+, TNBC) described in the result section and normal tissue. Hence, we used the BC081116e array. No stratification or matching was performed. The case collecting period and the follow-up period are not disclosed.

### 2.6. Immunohistochemistry

IHC staining of α-parvin (cat no. 11202-1-AP Proteintech, Rosemont, IL, USA [22]) was performed using the VECTASTAIN ABC Standard kit (cat no. PK-4000, Vector Laboratories Inc., Newark, CA, USA) following the manufacturer’s protocols. Briefly, the paraffin-embedded sections were deparaffinized, rehydrated, and heated at 105 °C for 15 min in HistoVT One pH 7.0 (cat no. 06380-76, Nacalai Tesque Inc.) for antigen retrieval. To inhibit endogenous peroxidase, the samples were immersed in a 3% hydrogen peroxide solution for 10 min at room temperature. Subsequently, the samples were washed with distilled water and TBS-T and then blocked with 5% goat serum TBS-T for 1 h. Following this, rabbit anti-α-parvin was applied at 1:500 dilutions and incubated overnight at 4 °C. After thorough washing, slides underwent incubation with biotinylated secondary antibodies and the streptavidin-biotin complex for 30 min each at room temperature, with washes in between. After washing, the slides were stained with ImmPACT 3,3′ diaminobenzidine (DAB) Substrate Kit (cat no. SK-4105, Vector laboratories Inc.) for 1 min, then rinsed with distilled water and counterstained with hematoxylin. The slides were coverslipped using Neo-Mount (109016, Merck KGaA, Darmstadt, Germany) and left in the dark to dry. Stained slides were converted to virtual slides, observed, and photographed with NDP view 2 (Hamamatsu Photonics, Shizuoka, Japan).

### 2.7. Image Analysis

HALO (Indica Labs, Albuquerque, NM, USA) was used for image visualization and analysis. All the cores were classified into three classes of cells (cancer, non-cancer, and stroma) using DenseNet V2 with a pathologist’s review. The staining status of each cell was evaluated using the Multiplex IHC v3.2.5 module. Cytoplasmic positivity was considered positive. DAB cytoplasm positive thresholds were 0, 0.1217, and 0.2655: negative, weak positive, moderate positive, and strong positive. Considering the percentage of cells at different intensity levels, the H-score was calculated with the formula: H-score = (percentage at weak positive) × 1 + (percentage at moderate positive) × 2 + (percentage at strong positive) × 3.

### 2.8. Statistical Analyses

R software (version 4.3.1) and Prism 10 (GraphPad) were used for the statistical analyses. Samples containing missing data were excluded from the analyses. The Mann–Whitney test was used for two-group comparisons, and the analysis of variance test and Dunn’s Multiple comparisons test were used for three or more group comparisons to calculate the respective *p*-values. In the correlation analysis, each variable in the TMA data was numerically converted to the following values for analysis. For ER and PR, “-” = 0, “+” = 1, “++” = 2, “+++” = 3. For HER2, “1+” = 1, “2+” = 2, “3+” = 3. For Ki67, “-” = 0, “+xx%” = xx. For stage, “IA” = 1, “IB” = 1.5, “IIA” = 2, “IIB” = 2.5, “IIIA” = 3, “IIIB, IIIC” = 3.5. For TNM, “T1NxMx” = 1, “T2NxMx” = 2, “T3NxMx” = 3, and “T4NxMx” = 4. Spearman’s rank correlation coefficient was employed for correlation plots, given the presence of ordinal variables in the dataset. No normality test was conducted, as the nonparametric nature of the method does not necessitate it. *p*-values less than 0.05 were considered significant. The following symbols were used for the respective significance levels: * *p* < 0.05, ** *p* < 0.01, *** *p* < 0.001, **** *p* < 0.0001.

## 3. Results

### 3.1. PARVA Amplification Is Frequently Observed in BC, and Its High Expression Is Associated with Poor Prognosis

**Figure 1:**
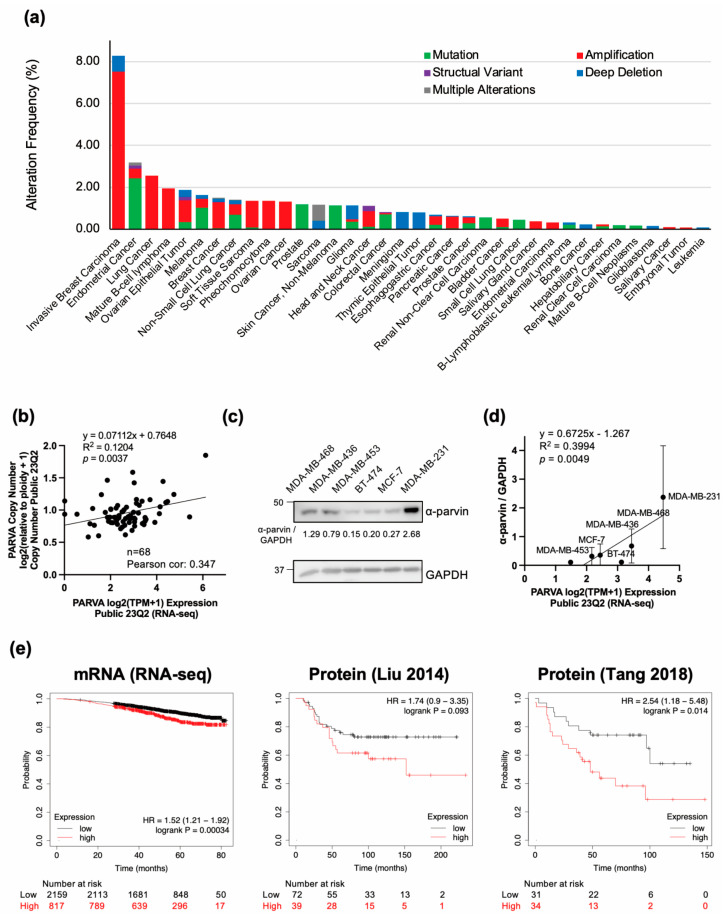
High levels of α-parvin associated with poor disease outcomes in BC. (**a**) cBioPortal shows increased PARVA amplification in human cancers. Cancers with >0.1% alteration frequency are shown. (**b**) Plot of BC cell lines for PARVA copy number and mRNA expression. Data were obtained from the DepMap portal. (**c**) Representative western blot (WB) data for α-parvin and GAPDH in 6 BC cell lines. α-Parvin expression levels were normalized by GAPDH, and the loading control and quantified values were shown under the α-parvin bands. (**d**) Plot of PARVA mRNA expression in DepMap portal versus α-parvin expression (*n* = 3). The dots indicate the mean of three WBs for each cell line, and the error bars represent SD. (**e**) Kaplan–Meier plot showing PARVA/α-parvin expression about BC patients’ overall survival rates. Two databases (Liu 2014 [26] and Tang 2018 [27]) were used to analyze protein expression. Abbreviations: BC, breast cancer; PARVA, gene encoding α-parvin; GAPDH, glyceraldehyde-3-phosphate dehydrogenase; SD, standard deviation.

To investigate its impact on various cancers, we analyzed the alteration frequency of the PARVA gene (α-parvin) using the publicly available database cBioportal. We evaluated 69,223 samples from 65,853 patients in 213 studies with PARVA gene information from all enrollment data sets without duplicate samples, including the Cancer Genome Atlas (TCGA) and non-TCGA studies. The findings revealed that PARVA amplification was prevalent in several human malignancies, with BCs having the highest prevalence (Figure 1a, Supplementary Materials Table S1). The most common changes in the PARVA gene in cancer were amplification, rather than mutation or deletion (Figure 1a, Supplementary Materials Table S1). Among BC types, breast sarcoma did not show PARVA amplification, although invasive breast cancer and BC did (Supplementary Materials Table S2). These findings suggest that amplification of the PARVA may be involved in BC.

Next, we analyzed the correlation between copy numbers and mRNA expression in BC cell lines. Of 1864 cancer cell lines from the public database DepMap portal (https://depmap.org/portal/ accessed on 1 August 2024), 68 were of BCs with information on copy number and RNA-seq data of PARVA (Supplementary Materials Table S3). The analysis showed that PARVA copy number and mRNA expression were positively correlated with Pearson’s correlation coefficient of 0.347 (Figure 1b). The regression coefficient also showed a significant positive correlation (*p* = 0.0037, Figure 1b), supporting an association between PARVA gene amplification and increased RNA. 

To investigate the relationship between PARVA mRNA and α-parvin expression in BC, protein expression levels were examined by IB in six TNBC cell lines. The results showed that α-parvin expression was exceptionally high in MDAMB231 cells with high RNA expression (Figure 1c and Supplementary Materials Table S3). The linear regression from the plot of the quantified α-parvin IB results (*n* = 3) against mRNA expression from DepMap in the six TNBC cell lines showed a significant increase (*p* = 0.0049) (Figure 1d, Supplementary Materials Table S3). Thus, our results indicate a positive correlation between the PARVA gene and its mRNA, and mRNA and protein expression in BC, suggesting that PARVA/α-parvin may contribute to BC.

To examine the impact of the expression of PARVA/α-parvin on the prognosis of BC patients, we generated survival curves. From the online platform Kaplan–Meier plotter, one dataset of PARVA mRNA expression levels measured by RNA-seq and two datasets (Liu et al. and Tang et al.) [26,27] of α-parvin expression were used to generate survival curves for two groups according to high and low PARVA/α-parvin expression. Results showed that high PARVA mRNA expression in patients with BC significantly contributed to worse overall survival. Similarly, the high α-parvin expression group showed a trend toward worse overall survival in both datasets, with the data of Tang et al. [27] being significant (Figure 1e). These results suggest that high expression of PARVA/α-parvin may be associated with pathogenesis and contribute to poor prognosis in BC.

### 3.2. Expression of α-Parvin Increased in BC Patients

**Figure 2:**
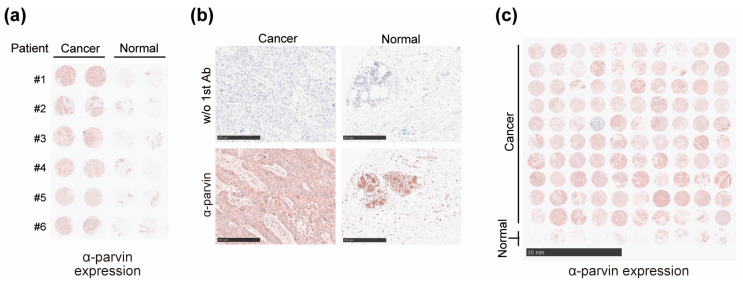
Immunohistochemical images of α-parvin expression in breast carcinoma (Cancer) and cancer-adjacent breast tissue (Normal). (**a**) α-Parvin-stained image of BR251f tissue microarray. Patients are indicated by number, and four cores from the same patient are shown on the same row (cancer *n* = 6, normal *n* = 6, quadruple cores per case). (**b**) Images of BR251f tissue microarray (patient #1) stained with α-parvin antibody or without 1st antibody. Scale bar: 250 µm (**c**) Entire α-parvin-stained image of BC081116e (cancer *n* = 100, normal *n* = 10). Scale bar: 5 mm.

To validate the in-silico results, we compared the expression of α-parvin in tissues of patients with BC with that in normal tissues by IHC. First, we stained BR251f TMA, which contained 24 cores with six cases representing breast carcinoma with a matched normal breast tissue array. Staining results showed higher expression of α-parvin in cancer tissues than in normal tissues, and a similar trend was observed in all six patients (Figure 2a). Expression of α-parvin was observed in normal and cancer tissues, with positive expression in epithelia and conduits (Figure 2b). Meanwhile, there were individual differences in α-parvin expression levels in the cancer tissues. α-Parvin expression might be elevated in stromal areas where it was low in normal tissues (Figure 2b). Recently, the role of stromal tissue in the development of BC has been considered crucial [28,29]. Among the biomarkers of stromal tissue implicated in BC, α-parvin may be a possible candidate. To investigate this result further, we performed a validation using another TMA, BC081116e, with a larger sample size. IHC staining results of BC081116e showed increased α-parvin expression in BC patients, similar to BR251f (Figure 2c).

### 3.3. α-Parvin Level Elevated in the Stromal Region in BC

To further study the expression of α-parvin in BC, we quantified the expression levels of each compartment using artificial intelligence (AI) and analyzed them according to their clinical subtype. BC081116e contained 110 cores with 107 cases representing 100 BC with 10 cancer-adjacent breast tissues with IHC information regarding ER, PR, HER2, and Ki67 and clinical information (pathology grade, TNM, and stage). The TMA includes at least ten cases of each clinical subtype and ten cores of adjacent tissue, allowing comparison of BC tissue with normal tissue. Initially, based on the pathologist’s observations, five of the 110 cores were excluded that were not appropriate for analysis. These included one cancer core with a mixture of tumor and non-tumor (No. 79), one that was considered a cancer core but was non-tumor (No. 83), two cores with detached tissue (Nos. 101 and 104), and one with only stroma and no cells (No. 106) (Supplementary Materials Figure S1). The basic statistics for the 105 cores used in the analysis are shown in Table 1.

**Figure 3:**
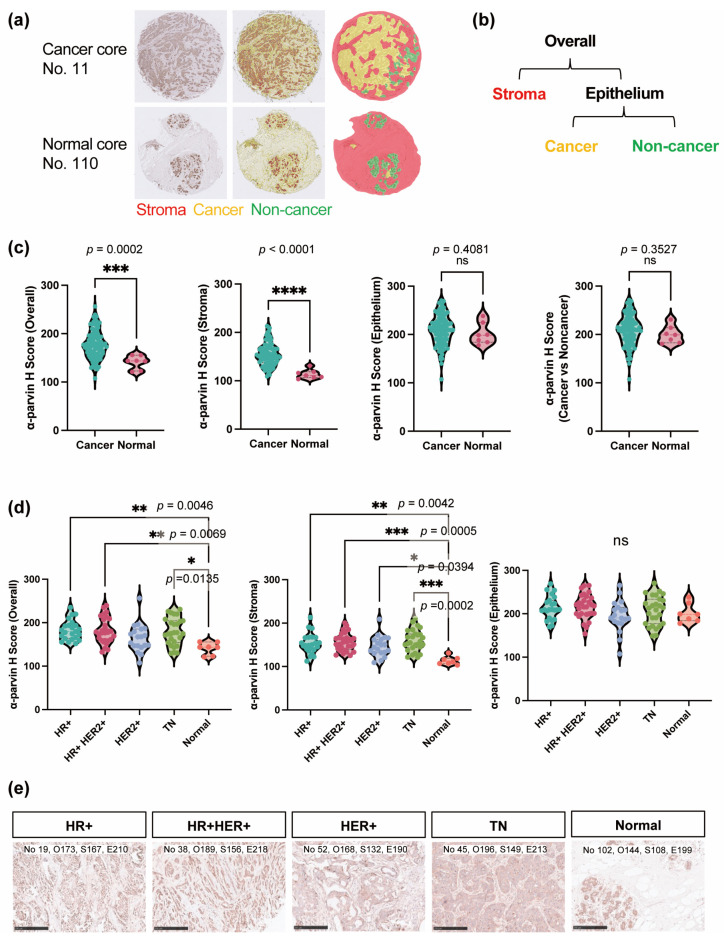
Expression of α-parvin was increased in the stroma region of BC. (**a**) Representative examples of post-learning images in HALO. The red area was recognized as stroma, yellow as cancer, and green as non-cancer. (**b**) Description of the three H-score areas. “Overall” indicates the entire core, “Stroma” indicates areas classified as stroma by HALO (red), and “Epithelium” displays the sum of areas classified as cancer (yellow) and non-cancer (green) by HALO. (**c**) The respective H-scores in the cancer and normal cores. The H-score (Cancer vs Noncancer) is the cancer area of cancer cores and the non-cancer area of normal cores. (**d**) Comparison of each H-score by clinical subtype: HR+ indicates ER-positive; HR+HER2+, ER- and HER2-positive; HER2+, HER2-positive; TN, triple negative; Normal, normal core. (**e**) α-Parvin immunostaining images of representative subtypes; No indicates ID; O, overall; S, stroma; and E, epithelial H-score. Scale bar: 250 µm. ns = Not Significant, * *p* < 0.05, ** *p* < 0.01, *** *p* < 0.001, **** *p* < 0.0001. Abbreviations: BC, breast cancer; ER, estrogen receptor; HER, human epidermal growth factor receptor; TN, triple negative; HR, hormone receptor.

The pathologist then annotated representative stroma, cancer, and noncancer cells to train the program as described in Section 2, and all cores were designated into these areas (Figure 3a and Supplementary Materials Figure S2). To compare the expression of α-parvin in each compartment of BC between normal cores, we first calculated four H-scores (overall, stroma, epithelium, and cancer vs noncancer) using HALO-based regional classification (stroma, cancer, non-cancer). The “overall” H-score is calculated as the entire core, the “stroma” H-score is calculated as the area classified as stroma by HALO (red), the “epithelium” H-score is the sum of areas classified as cancer (yellow) and the non-cancer (green) by HALO, and “cancer vs non-cancer” H-score is the cancer area of cancer cores and the non-cancer area of normal cores (Figure 3b, Supplementary Materials Table S4). A comparison of H-scores between cancer and normal cores showed significantly higher α-parvin expression in the overall and stroma of BCs (Figure 3c), but no significant difference in the elevated expression of α-parvin in epithelial and cancer vs noncancer scores (Figure 3c). Thus, there was no significant difference in α-parvin expression between cancer and normal cells in the epithelium. Since the calculations showed that the scores of epithelia and cancer vs noncancer were almost identical, the epithelium, which encompasses both regions, was used in further analyses.

### 3.4. All BC Subtypes Had Elevated α-Parvin Expression Levels in Stroma

BC is classified according to the presence or absence of hormone receptors (ER and PR) and HER2. They have been categorized and treated with respective targeted drugs in recent years [2,3,4]. BC prognosis varies by subtype [5,6,7]. Among the subtypes, TNBC, which accounts for 12–20% of BC patients [6,8,9], has a worse prognosis than other clinical subtypes [5,6,7]. Targeted therapy has improved other subtypes’ prognoses, but their 5-year survival rates are not 100% [30]. Hence, examining the expression of α-parvin in each subtype is valuable.

Therefore, to analyze the differences in α-parvin expression by subtype, we performed an intergroup comparison of the H-scores of each core. The results showed no significant changes by clinical subtype, indicating higher overall and stromal α-parvin expression than the normal core (Figure 3d). The representative images and H-scores of each clinical subtype are shown in Figure 3e. These results indicate that α-parvin is highly expressed in the stroma of BC, regardless of hormone receptors or HER2 expression.

### 3.5. α-Parvin Expression in BC Correlates Positively with Ki67, While That in the Stroma Correlates with Grade, TNM, and Stage

**Figure 4:**
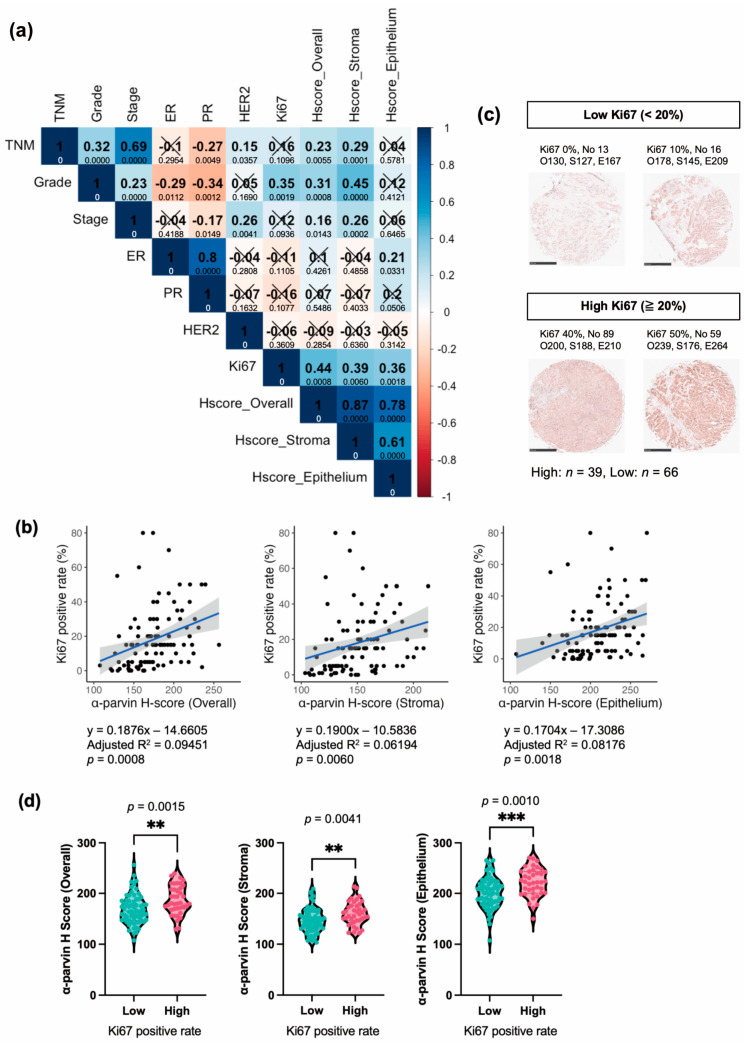
Expression of α-parvin in BC was positively correlated with Ki67. (**a**) Correlation plots between breast cancer (BC) factors and each H-score. Each number in the center indicates Spearman’s correlation coefficient, and *p*-values are shown below each number. ‘×’ indicates no significance. (**b**) Linear regressions of Ki67 positive rate and H-scores. The respective regression equations, coefficients of determination, and *p*-values are shown below the figure. (**c**) α-Parvin immunostaining images of different Ki67 positive rate; No indicates ID; O, overall; S, stroma; and E, epithelial H-score. The number of cores for each group is shown below. Scale bar: 500 µm. (**d**) Comparison of H-scores by high and low Ki67 positive rate. ** *p* < 0.01, *** *p* < 0.001.

To determine the relationship between α-parvin expression and clinical factors of BC, we performed correlation analysis between H-scores, Ki67, TNM, pathology grade, and stage in addition to ER, PR, and HER2 in BC081116e. The results showed a significant positive correlation of α-parvin with Ki67 in overall, stroma, and epithelium (correlation coefficients: 0.36–0.44), suggesting an association of α-parvin with BC cell proliferation. In addition, α-parvin in the overall and stroma was significantly positively correlated with grade and stage, as well as TNM (correlation coefficients: 0.16–0.45). Conversely, no significant correlation between α-parvin and the hormone receptors ER, PR, and HER2 was observed. However, only epithelial α-parvin showed a positive correlation with ER (correlation coefficient: 0.21) (Figure 4a). The Ki67 positivity rate and respective H-scores were plotted for BC cores, and the linear regression models and equations are shown in Figure 4b. The results show a significant increase in Ki67 as all H-scores increase. 

For a detailed study, we analyzed the expression levels of α-parvin according to the high and low Ki67 positive rates. Although the criteria for high and low Ki67 positive rates vary, 15–30% is often set in many studies and clinical fields [31,32]. Based on these studies [31], we used 20% as the standard for our analysis. The low group with <20% Ki67 positive rate had 66 cores, and the ≥20% group had 39 cores. IHC images of representative cores by Ki67 high and low are shown in Figure 4c. A subsequent comparison of H-scores by Ki67 high and low resulted in significantly higher H-scores in the high Ki67 group for overall, stroma, and epithelium (Figure 4d). 

Thus, our results indicate that α-parvin is not only elevated in the stroma but is also associated with Ki67 in the epithelium and may contribute to BC pathology.

## 4. Discussion

This study suggests that α-parvin likely contributes to BC pathology. The in-silico analysis showed that amplification of PARVA and high expression of α-parvin may play a prognostic role in BC (Figure 1). IHC and HALO analysis, an objective quantitative assessment of α-parvin levels in different cancer tissue compartments in BC patients, showed a significant increase in α-parvin levels in the stromal region of cancer tissue compared to adjacent tissue (Figure 3c). There was a significant increase in α-parvin levels in the stroma in all BC subtypes (Figure 3d). In addition, correlation analysis with indicators of BC showed a significant positive correlation of α-parvin with Ki67 (Figure 4a). These results suggest that α-parvin may be a potential biomarker highly expressed in BC patients, especially in the stroma, and may contribute to establishing new diagnostic and therapeutic methods.

The mechanisms by which the stroma promotes tumor progression have been described earlier, in which the involvement of alterations in integrin signaling in fibroblasts was suggested [28,29]. Accumulating evidence demonstrated that α-parvin facilitates cellular adhesion, cytoskeleton reorganization, and cell survival via the integrin signaling pathway, and its deregulation is associated with cancer [33]. Therefore, increased α-parvin expression in BC stromal cells, particularly BC fibroblasts, could lead to a poor prognosis by altering integrin signaling to enhance tumor progression. In addition, α-parvin participates in multiple signaling pathways to regulate metastasis and invasion [34]. In lung cancer progression, α-parvin acts as an oncogene by activating Akt and inhibiting GSK3β [19]. It is important to elucidate how increased α-parvin expression in BC stromal cells affects the behavior of cancer cells and/or the tumor microenvironment and clarify the underlying molecular mechanisms.

Our finding that overall α-parvin expression was increased in BC aligns with a previous report [21]. Furthermore, through objective assessment and compartmentalization using an imaging AI, HALO, we demonstrated increased α-parvin expression in the stromal region but not in the epithelial region between BC and adjacent tissues (Figure 3c), suggesting that elevated α-parvin levels in the stroma are responsible for increased α-parvin levels in BC. In contrast to our finding in BC tissues, downregulation of α-parvin expression was reported in prostate cancer [22]. Therefore, the effects of α-parvin overexpression or downregulation in cancer progression could be different among cancer types; this needs to be clarified by precise analysis of α-parvin expression in tumor cells and stromal cells.

High expression of α-parvin in BC tissue as a whole and in the stroma was found in all BC subtypes compared to normal tissue (Figure 3d). There were no significant differences in α-parvin levels between TNBC, HR-positive BC, and HER2-positive BC (Figure 3d). Our results differed from a previous study with BC cell lines which reported significantly higher α-parvin levels in TNBC cell lines than HR-positive and HER2-positive cell lines. This may be due to differences in cell lines and tissues, as cell lines do not contain stroma, which may have led to different results. Thus, our findings suggest that both the epithelium and stromal regions are essential for understanding BC.

Both the overall and stromal α-parvin H-scores were not correlated with HR or HER2 expression but were significantly positively correlated with TNM, stage, and grade of BC (Figure 4a). These findings suggest that high expression of α-parvin in the stroma may play a role in tumor size, cancer progression, and pathological grade. Therefore, suppressing or inhibiting α-parvin expression could be a new targeted therapeutic strategy. Indeed, previous studies have reported that α-parvin deficiency inhibits BC growth [21] and that its expression contributes to metastasis; overexpression of α-parvin in the BC cell lines increased transmigration and migration, which were decreased by siRNA knockdown [20]. In addition to BC, overexpression of α-parvin promotes colony formation and tube formation in lung adenocarcinoma cell lines and promotes angiogenesis in mice [19]. It has also been reported that overexpression of α-parvin increased invasion in melanoma cell lines and tumor volume of the cells in mice, both suppressed by knockdown of α-parvin [35]. These studies support the possibility that increased expression of α-parvin at the cellular-to-individual level promotes cancer. 

ILK, a component of the IPP complex, was also reported to contribute to metastasis and EMT [36]. As another example, modulation of the cell-ECM adhesion protein Ras Suppressor-1 (RSU-1) may promote BC metastasis; RSU-1 interacts with PINCH-1, a component of the IPP complex, and plays an essential role in the invasion by associating with ILK and α-parvin [37]. The increased expression of the scaffolding IPP complex may have significantly impacted the signaling changes. Further studies are needed to identify signaling pathways and the contribution of other components of the IPP complexes to BC.

In this study, there is the possibility of selection bias due to the unavailability of the patient background of the samples. In order to analyze the potential use of parvin as a biomarker to predict the prognosis and treatment effect, it is necessary to use more clinical data such as the survival rate and treatment of each patient. Future analysis of clinical data and parvin protein levels will allow us to propose guidelines for use as a new biomarker for breast cancer. In addition, IHC standardization using automated immunostaining equipment would be necessary for practical use. The role of α-parvin should be further investigated in more extensive and different samples of BC. In addition, it is important to examine the effect of α-parvin on the treatment response of patients. Also, the study of other cancer types or in vitro and in vivo functional studies of α-parvin is needed. In future research, it will be necessary to identify which stromal cells exhibit elevated α-parvin expression through co-staining with markers. 

Our results showed that all H-scores, overall, stroma, and epithelium scores, were significantly positively correlated with Ki67, a proliferation marker (Figure 4a). Also, BCs that expressed high α-parvin showed a significantly higher positivity rate of Ki67 (Figure 4d). Because Ki67 is an indicator of BC proliferation and is important in determining whether chemotherapy should be administered [32,38], our finding suggests that α-parvin could be a marker for deciding on the use of chemotherapy, and α-parvin may be a potential therapeutic target for BC with high cell proliferative potential. It may also be a new target for currently untreatable BC.

## Figures and Tables

**Table 1:** Basic statistics of the samples on the BC081116e. The data was obtained from TissueArray.com LLC.

Characteristic	*N* = 105 ^1^
Age	47 (10)
Sex	
Female	105/105 (100%)
Type	
AT	7/105 (6.7%)
Malignant	98/105 (93%)
TNM	
−	7/105 (6.7%)
T1NxMx	2/105 (1.9%)
T2NxMx	75/105 (71%)
T3NxMx	14/105 (13%)
T4NxMx	7/105 (6.7%)
Grade	
−	8/105 (7.6%)
1	3/105 (2.9%)
2	62/105 (59%)
3	32/105 (30%)
Stage	
−	7/105 (6.7%)
IA	1/105 (1.0%)
IIA	49/105 (47%)
IIB	22/105 (21%)
IIIA	18/105 (17%)
IIIB	7/105 (6.7%)
IIIC	1/105 (1.0%)
ER	
−	46/105 (44%)
+	15/105 (14%)
++	9/105 (8.6%)
+++	35/105 (33%)
PR	
−	57/105 (54%)
+	14/105 (13%)
++	11/105 (10%)
+++	23/105 (22%)
HER2	
0	57/105 (54%)
1+	13/105 (12%)
2+	12/105 (11%)
3+	23/105 (22%)
Ki67	18 (18)
Subtype	
HR+	23/105 (22%)
HER+	19/105 (18%)
HR+ HER+	29/105 (28%)
TN	27/105 (26%)
Normal (AT)	7/105 (6.7%)
Pathology diagnosis	
Invasive carcinoma of no special type	97/105 (92%)
Breast carcinoma with apocrine differentiation	1/105 (1.0%)
Cancer adjacent breast tissue	7/105 (6.7%)

^1^ Mean (standard deviation [SD]); *n*/*N* (%); TNM, tumor, node, and metastasis; ER, estrogen receptor; HER, human epidermal growth factor receptor; PR, progesterone receptor; TN, triple negative; “AT” indicates cancer adjacent breast tissue. “−” indicates a negative for the variables. Subtype was classified according to ER and HER2 status: positive (+ or 1+ or higher) or negative (− or 0).

## Data Availability

The data that support the findings of this study are available from the author, M.K., upon reasonable request.

## Supplementary Materials

The following supporting information can be downloaded at: https://www.mdpi.com/article/10.3390/cells13181572/s1, Figure S1: IHC stained images of excluded cores; Figure S2: Enlarged image of Figure 3a; Table S1: PARVA gene alteration frequency by cancer study; Table S2: PARVA gene alteration frequency by breast cancer type; Table S3: Breast cancer cell lines with PARVA copy number and mRNA information; Table S4: List of clinical information and H-scores for BC081116e.
