# Supplementary figures and images for: α-Parvin Expression in Breast Cancer Tissues: Correlation with Clinical Parameters and Prognostic Significance
Source: Cells. 2024 Sep 19;13(18):1572. doi: 10.3390/cells13181572 (PMC11430769; doi:10.3390/cells13181572)

## $\alpha$ -parvin expression

---

Mixed tumor and  
non-tumor (No 79)

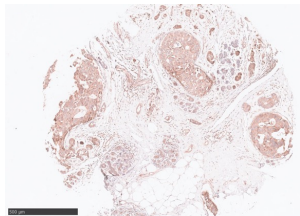

Non-tumor (No 83)

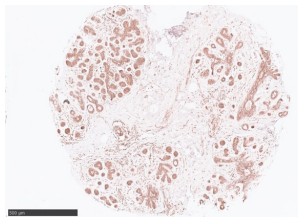

Peeled off (No 104)

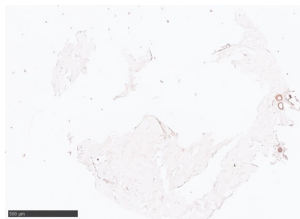

No cells (No 106)

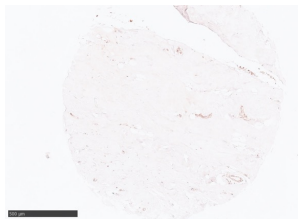

Supplement: Supplementary file 1 [file cells-13-01572-s001.zip › Supplementary figure S1.pdf]
